# *Experimental Physiology* Special Issue: Exercise as Medicine

**DOI:** 10.1113/EP093924

**Published:** 2026-05-27

**Authors:** Ronan M. G Berg, Colleen S Deane, Jill N Barnes, Harry B Rossiter

**Affiliations:** ^1^ Centre for Physical Activity Research Copenhagen University Hospital – Rigshospitalet Copenhagen Denmark; ^2^ Department of Clinical Physiology and Nuclear Medicine Copenhagen University Hospital – Rigshospitalet Copenhagen Denmark; ^3^ Department of Clinical Medicine, Faculty of Health and Medical Sciences University of Copenhagen Copenhagen Denmark; ^4^ Neurovascular Research Laboratory, Faculty of Life Sciences and Education University of South Wales Pontypridd UK; ^5^ Human Development & Health, Faculty of Medicine, Southampton General Hospital University of Southampton Southampton UK; ^6^ National Institute of Health Research (NIHR) Southampton Biomedical Research Centre (BRC) University Hospital Southampton NHS Foundation Trust and University of Southampton Southampton UK; ^7^ Bruno Balke Biodynamics Laboratory University of Wisconsin‐Madison Madison Wisconsin USA; ^8^ Respiratory Research Center The Lundquist Institute for Biomedical Innovation at Harbor‐UCLA Medical Center Torrance California USA; ^9^ Division of Respiratory and Critical Care, Physiology and Medicine, Department of Medicine Harbor‐UCLA Medical Center Torrance California USA

A defining feature of *Exercise as Medicine* is that it concerns the use of exercise as a treatment in individuals who have already developed disease, rather than exercise as an end in itself or as a primary preventive strategy (Febbraio & Pedersen, 2026). This concept should not be confused with Exercise is Medicine®, a registered global health initiative launched in 2007 by the American College of Sports Medicine in partnership with the American Medical Association. The Exercise is Medicine® initiative primarily aims to address physical inactivity by encouraging clinicians to promote or prescribe exercise within routine care pathways (Sallis, 2009). By contrast, *Exercise as Medicine* is not an advocacy programme, but rather denotes a transdisciplinary research framework in which exercise, a subset of physical activity that is planned and structured to achieve a final or intermediate objective of gaining health benefits associated with improving or maintaining physical fitness (Craighead et al., 2026), is explicitly conceptualised as a therapeutic intervention for established disease, spanning work from mechanistic studies to clinical randomised controlled trials (Febbraio & Pedersen, 2026; Pedersen, 2019). Here, ‘medicine’ is broadly defined as an intervention used to prevent, diagnose, treat or relieve symptoms of a disease or abnormal condition, without necessarily altering its progression. Within this framework, exercise is evaluated using the same principles applied to other medical treatments, including indication, dose, efficacy, mechanisms of action and safety. The deliberate integration of mechanistic and clinical evidence is a fundamental feature of this approach (Berg et al., 2025). Mechanistic studies are thus used to investigate whether and how exercise affects multiple biological pathways and inter‐organ signalling, providing causal explanations for clinically meaningful effects on disease progression and outcomes (Chow et al., 2022; Severinsen & Pedersen, 2020) whereas adequately designed clinical trials are required for the evaluation of potential harms and contraindications relative to clinical benefits, with the same rigour as for pharmacological treatments. Consequently, exercise is not automatically indicated for all disease conditions and should only be recommended when supported by the best available evidence and applied with appropriate clinical judgement.

As a concept, *Exercise as Medicine* appears to date back almost as far as clinical medicine itself (Berryman, 2010; Febbraio & Pedersen, 2026; Tipton, 2014). Hippocrates (c. 460–370 BC) emphasised the therapeutic importance of physical activity, stating that ‘eating alone will not keep a man well; he must also take exercise’ (Tipton, 2014). The idea was reiterated in several classical medical writings across the subsequent two millennia, including Galen of Pergamon (129–c. 216) in *De sanitate tuenda* (On the Preservation of Health), Cristóbal Méndez (c. 1500–1556) in *Libro del ejercicio corporal y de sus provechos* (Book of Bodily Exercise, 1553), Francis Fuller (1670–1706) in *Medicina Gymnastica: Or, a Treatise Concerning the Power of Exercise* (1705), and William Buchan (1729–1805) in *Domestic Medicine*. In these accounts, medicine was not yet a modern science; instead, it was a craft rooted in natural philosophy and anecdotal experience rather than systematic experimentation.

A turning point in the history of physiology and medicine was the work of William Harvey (1778–1657), who described in *de Motu Cordis* (1628) the first true experimental methods showing the link between structure to function in proving that blood circulates through the body in a closed system pumped by the heart. In the nineteenth century, this experimental approach propelled physiology as the study of how living systems function, which emerged as a dedicated and independent experimental science that, together with pathology and bacteriology, informed clinical medicine (Cunningham, 2002, 2003). Accordingly, Carl Ludwig (1816–1895), a key figure in this transition and often regarded as the father of modern physiology, argued that the purpose of physiology was ultimately ‘to serve the clinics’ (Schubert, 1996). Even so, exercise initially served primarily as a physiological model or controlled stressor to physiological systems, as a means of provoking measurable changes to reveal the underlying principles of bodily regulation and adaptation. During the early twentieth century, and particularly in the interwar period, the scientific study of exercise gradually developed into a more distinct field within physiology. In his book *Features in the Architecture of Physiological Function* (1934), the British physiologist Sir Joseph Barcroft (1872–1947) argued that there was a ‘resting state bias’ in physiological research and that the human body could not be truly understood by studying it at rest. He argued that it was like studying a car with the engine turned off: you can see the parts, but you cannot understand what they are *for*. His thesis that ‘exercise is the essence of the machine’ was built on the notion that the function of the pulmonary, circulatory and neuromuscular systems had evolved to meet the demands of physical exertion and therefore studying the physiology of these systems only made sense when the body was considered in motion.

Early twentieth century investigations of exercise were often motivated by concerns regarding the potential harms of intense athletic performance, but they increasingly expanded to address the broader health consequences of habitual physical activity and inactivity. At the same time, the post‐World War II emergence of clinical epidemiology provided complementary evidence associating habitual physical activity with disease risk. For example, a seminal series of studies, sometimes informally referred to as the London Busmen Study, reported that bus conductors had a lower risk of dying from ischaemic heart disease than the more sedentary bus drivers (Heady et al., 1961; Morris & Raffle, 1954). These observations were subsequently followed by numerous epidemiological studies identifying physical inactivity as a risk factor for a wide range of chronic diseases. Parallel developments within the field of experimental physiology further clarified the physiological consequences of inactivity. The Dallas Bedrest and Training Study for one, demonstrated the detrimental effects of prolonged bed rest (Saltin et al., 1968), which, at that time, was commonly prescribed for several conditions, including recovery after myocardial infarction. The study showed marked reductions in cardiac function and aerobic capacity following 3 weeks of strict bedrest, changes that were only partially reversed by subsequent intense exercise training. The post‐World War II era also witnessed the introduction of formal randomised controlled trials evaluating exercise interventions in specific disease states (Sanne, 1973; Taylor et al., 1973). Collectively, these developments across the twentieth century laid the empirical foundations for the modern concept of *Exercise as Medicine*. As the Danish physiologist Erling Asmussen (1907–1991) remarked in his opening address at the symposium *Physiology of Muscular Exercise* in Dallas (Asmussen, 1967): ‘It is extremely important to know how systematic physical exercise, or lack of exercise, influences the growing child, the young adult, and the elderly, with respect to his or her powers to resist or recover from diseases, accidents, and degenerative changes … The medical profession, the single individual, and society would like to be better informed.’

Asmussen's sentiment clearly still resonated at the turn of the millennium when the Danish Health Authority commissioned Bengt Saltin (1935–2014), the principal investigator of the Dallas Bedrest and Training Study, and Bente Klarlund Pedersen to produce an extensive report synthesising the available evidence on exercise as a therapeutic intervention across a range of diseases (Pedersen & Saltin, 2003), and their peer‐reviewed findings were later published in English in 2006 (Pedersen & Saltin, 2006). This review evaluated exercise therapy across 18 diseases and conditions spanning metabolic, cardiopulmonary, musculoskeletal and other clinical categories. The authors systematically assessed the effects of exercise on disease pathogenesis, diagnosis‐specific symptoms, physical fitness or strength, and quality of life. Potential mechanisms of action were considered, and practical principles for prescribing exercise therapy were discussed, including exercise modality, volume and possible contraindications. An updated version published 9 years later expanded the scope to 35 diseases and conditions (Pedersen & Saltin, 2015); together, these two reviews have been cited more than 7000 times. Since then, numerous systematic reviews and meta‐analyses have further evaluated the therapeutic effects of exercise across clinical populations. For example, a recent overview of systematic reviews covering the period from 2000 to 2023 identified evidence across 45 diseases (Dibben et al., 2024). Collectively, the evidence synthesised over the past two decades supports the use of exercise therapy across a wide range of chronic diseases, with exercise increasingly framed in terms analogous to other medical treatments, including therapeutic indication, dose, efficacy and safety. The strength and nature of evidence for exercise as medicine vary across conditions, and there remains a lot of work to do, especially in delivering randomized controlled trials to investigate potential disease‐modifying benefits of exercise. In many cases, robust evidence exists for improvements in cardiorespiratory fitness, functional capacity and symptom burden and in some cases even mortality, but evidence for clear dose–response relationships and disease‐modifying effects is more limited and disease‐specific.

This Special Issue of *Experimental Physiology* reflects the breadth of *Exercise as Medicine* and its inherently translational nature, spanning research from mechanistic studies to clinical trials. In addition to two accompanying editorials (Craighead et al., 2026; Hartmann et al., 2026), and a viewpoint (Schulze & Poole, 2026), the issue includes 14 original research articles and nine review articles, as well as one Letter (Hagar, 2026) and one Registered Report (Tavares et al., 2026) as outlined below. The latter forms part of *Experimental Physiology*’s initiative to address ongoing challenges regarding the robustness and replicability of reported findings within the field (Rasmussen et al., 2025).

Craighead and colleagues contribute an editorial that discusses basic conceptual distinctions within the field of *Exercise as Medicine*, including the relationship between ‘exercise’, ‘physical activity’ and related terms (Craighead et al., 2026), building on the classical and widely cited framework proposed by Caspersen and colleagues (1985).

Several papers in this Special Issue address exercise limitation, physiological adaptation and their assessment in various lung diseases. In chronic obstructive pulmonary disease (COPD), Abbasi et al. investigate the response of immune cells and inflammatory markers to exercise training in the context of cigarette smoke exposure (Abbasi et al., 2026). Their findings indicate that exercise training modifies pulmonary inflammation and slows the development or progression of emphysema in smoke‐exposed mice; however, evidence remains limited to support comparable immunomodulatory effects of pulmonary rehabilitation in patients with established COPD. Other contributions focus on the physiological determinants of exercise limitation in COPD within the framework of the oxygen transport cascade. Rasmussen et al. demonstrate that assessment of pulmonary alveolar–capillary reserve in the supine position yields information consistent with measurements obtained during exercise testing (Rasmussen et al., 2026b), and find no evidence of specific pulmonary exercise training adaptations, consistent with recent findings using exercise‐based approaches (Hartmann et al., 2025). These studies underscore the considerable importance of peripheral exercise adaptations in COPD, a topic discussed in detail (Hartmann et al., 2026; Schulze & Poole, 2026). In addition, one study critically evaluates the test–retest reliability of Doppler ultrasound‐based measurements of femoral blood flow during knee‐extensor exercise in patients with COPD compared with matched healthy controls (Mohammad et al., 2026). This approach represents a widely investigated method that aims to increase the volume of leg muscle engaged in exercise above that achievable with whole body exercise in patients who are limited by their breathing mechanics and therefore promote greater peripheral skeletal muscle adaptations to exercise training.

The interaction between pulmonary disease and locomotor muscle function is further explored in fibrotic interstitial lung disease (ILD). Thivent *et al*. show that ILD is characterised by several factors known to promote skeletal muscle dysfunction and provide new evidence supporting a role for locomotor muscle impairment in exercise limitation (Thivent et al., 2026). Notably, the authors report that exercise training programmes currently offered to these patients result in minimal and inconsistent gains in muscle strength, highlighting the need for alternative strategies to facilitate effective training and address existing impairments. Also, methodological considerations related to exercise testing in patients with severe pulmonary disease are addressed. Because maximal or all‐out exercise tests may be challenging to perform in individuals with COPD, alternative testing strategies are required. De Brandt *et al*. explore the use of the modified Borg cycle strength test in people with COPD and show high feasibility in this population. The approach allows power outputs exceeding 150% of those at peak oxygen uptake to be achieved, while eliciting physiological responses similar to those observed in healthy older adults (De Brandt et al., 2026).

A study focusing on a thematically related concept examines the role of exercise in preserving vitality capacity during ageing – defined by WHO as one of the five components of all physical and mental capacities an individual possesses (Jones et al., 2026). Ageing is a complex and multifaceted process associated with progressive deficits across multiple components contributing to vitality capacity. However, regular exercise can induce physiological adaptations that may be beneficial in the context of healthy ageing, potentially improving or preserving the status of these components. Respiratory muscle endurance training represents another intervention explored in this Special Issue (Laurent et al., 2026). Such training is reported to increase respiratory endurance; however, the populations that benefit most and the underlying mechanisms remain to be clarified. It also remains uncertain whether respiratory muscle endurance training should be performed as a stand‐alone intervention or as an adjunct to established pulmonary rehabilitation programmes. Following up on previous work demonstrating that 12 weeks of supervised high‐intensity interval training preserves cardiac mass after COVID‐19 (Rasmussen et al., 2023), the study presented in this Special Issue shows that this effect appears to persist at 12‐month follow‐up (Rasmussen et al., 2026a).

Another theme represented in this Special Issue concerns cardiovascular physiology. One study examines whether essential hypertension influences exercise‐induced cardiometabolic adaptations and whether the altered blood pressure response observed in individuals receiving antihypertensive medication modifies these benefits. Fischer et al. report that males with essential hypertension, whether treated or untreated, exhibit smaller improvements in cardiac function and cardiorespiratory fitness following high‐intensity interval training compared with normotensive individuals (Fischer et al., 2026). Notably, the findings raise the possibility that antihypertensive medication may interfere with potentially beneficial cardiac adaptations to exercise, an issue that warrants further investigation in prospective clinical trials. Another study examines microRNA‐126, one of the most abundantly expressed microRNAs in endothelial cells, which is known to be lower in individuals with prediabetes and type 2 diabetes (Ayaz et al., 2026). Here, 12 weeks of moderate‐intensity aerobic exercise training is reported to increase circulating microRNA‐126 levels in individuals with prediabetes, concomitant with favourable changes in markers of vascular function, including reduced carotid stiffness and an increased ankle–brachial index. Two narrative reviews further address vascular physiology in the context of female biology. One of these considers vascular adaptations to exercise across the female lifespan, emphasising that females experience substantial fluctuations in sex hormones associated with puberty, pregnancy and menopause, each of which influence vascular structure and function (Miller et al., 2026). Although habitual exercise is associated with reduced cardiovascular disease risk in women of all ages, the review highlights considerable variability in vascular adaptations to exercise, which may in part reflect these hormonal fluctuations. The other review focuses specifically on menopause and provides an extensive overview of vascular and physiological adaptations to exercise during this stage of life (Shing et al., 2026).

Female health is also addressed in an experimental study examining cerebrovascular and cognitive responses to two acute exercise interventions (Le Bourvellec et al., 2026). Acute exercise is known to enhance prefrontal cortex oxygenation and cognitive performance in young adults; however, whether similar benefits occur in postmenopausal women remains uncertain. Here it is found that neither acute isometric resistance exercise nor acute high‐intensity interval aerobic exercise improves cognitive performance in postmenopausal women, even though both interventions increase prefrontal cortex oxygenation during and following exercise.

In the area of endocrinology and glucose homeostasis, Morales et al. investigated the effects of a brief bout of physical activity on postprandial glycaemia (Morales et al., 2026). The authors report that a 1 min bout of self‐selected, low‐intensity stair stepping performed in the evening reduces the change from baseline to the 60 min time point in postprandial blood glucose, thereby lowering evening postprandial glucose levels in young, non‐diabetic adults. These findings suggest a promising and easily applicable behavioural strategy for improving glycaemic control, which may also prove beneficial in individuals with manifest type 2 diabetes. Another study examined whether brief 3 min bouts of resistance exercise, consisting of squats, high knees and calf raises – sometimes informally referred to as ‘exercise snacks’ – performed every 30 min during otherwise sedentary behaviour could augment the vasodilatory response to a subsequent oral glucose load (Rogers et al., 2026). This outcome is physiologically relevant because postprandial vasodilation reflects insulin‐mediated skeletal muscle perfusion, which facilitates glucose uptake and may represent an early marker of sedentary‐induced impairment of glucose homeostasis. The authors demonstrate that such intermittent exercise breaks improve the vasodilatory response compared with uninterrupted sitting.

Within the locomotor domain, several contributions address the role of exercise in musculoskeletal conditions. Sobral de Oliveira‐Souza et al. conducted a systematic review and meta‐analysis to evaluate the potential analgesic role of aerobic exercise in the treatment of neck pain (Sobral de Oliveira‐Souza et al., 2026). They found that aerobic exercise had some positive effects to reduce pain intensity, disability and emotional status but, the evidence is limited, low‐quality and heterogeneous. Notably, combining aerobic exercise with other therapeutic approaches appears more effective for reducing pain intensity than aerobic exercise alone. Knee osteoarthritis is addressed in another review, which highlights the complex relationship between physical activity and joint health (Morgan et al., 2026). Although physical activity is known to reduce knee pain, improve functional capacity and enhance quality of life, many individuals with knee osteoarthritis believe that exercise may cause further joint damage. The authors emphasise that a more nuanced understanding of the relationship between physical activity and knee osteoarthritis is required, both to address persistent misconceptions and to guide future research in this area. Another contribution examines cross‐education, a training phenomenon in which exercise of one limb induces strength gains in the contralateral, untrained limb (Altheyab et al., 2026). The proposed mechanisms underlying cross‐education are predominantly neural and include both the ‘cross‐activation’ hypothesis, reflecting a spillover of neural drive to the untrained limb, and the ‘bilateral‐access’ hypothesis, whereby central adaptations developed during unilateral training can be accessed by the contralateral limb (Carroll et al., 2006). This approach is of particular interest in individuals with temporarily immobilised limbs, for example during limb casting or following neurological injury such as stroke. The review reports that resistance exercise training can improve muscle strength in the untrained lower limb, with eccentric resistance exercise producing particularly pronounced increases in isometric peak torque. The available evidence further suggests that females may experience greater cross‐education effects than males with respect to strength adaptations; while this may be related to factors such as oestrogen‐mediated influences on neural adaptation (Altheyab et al., 2026), this finding is based on limited evidence and the underlying mechanisms remain unclear.

Another study explores exercise responses in individuals with persistent post‐concussion symptoms (Javra et al., 2026). The authors examined the feasibility, safety and physiological effects of combining head‐up tilt with lower body negative pressure during supine cycling. This approach aims to attenuate the increase in cerebral blood velocity typically observed during moderate‐intensity exercise in this population. The intervention is physiologically relevant because exercise‐induced hyperpnoea increases cerebral blood flow, which may exacerbate symptoms in individuals with post‐concussion syndrome due to intracranial pressure constraints. The study demonstrates that the addition of lower body negative pressure attenuates the hyperpnoea‐related increase in cerebral blood flow, while allowing participants to achieve higher heart rates without increasing perceived exertion and with fewer post‐exercise symptoms.

Three studies in this Special Issue address questions within exercise oncology. Neuberger et al. investigated how acute exercise affects the release and clearance of circulating cell‐free DNA in patients with solid tumours (Neuberger et al., 2026). The authors report that incremental exercise leads to an immediate increase in circulating cell‐free DNA and DNase I activity. However, these responses show less fluctuation than those observed in healthy individuals, highlighting the importance of considering recent exercise as a preanalytical factor when interpreting cancer liquid biopsy results. In a randomised controlled trial in men with treatment‐naïve localised prostate cancer, Thomsen et al. examined whether exercise training alters intratumoural immune cell composition. The study found no effect of the exercise intervention on CD3^+^ or CD8^+^ T‐cell density within the tumour microenvironment, suggesting that the training programme did not modify these aspects of tumour‐associated immune infiltration (Thomsen et al., 2026). However, given that previous preclinical studies suggest exercise may influence the balance between cytotoxic CD8^+^ T cells and immunosuppressive FoxP3^+^ regulatory T cells within the tumour microenvironment, further work is required to determine whether exercise influences this immunological balance in prostate cancer (Hagar, 2026). Finally, Tsitskanou et al. investigated whether colon cancer affects skeletal muscle adaptations to short‐term exercise training (Tsitkanou et al., 2026). Using a mouse model in which colon‐26 adenocarcinoma cells were inoculated to induce tumour growth, the authors report that skeletal muscle adaptations in the gastrocnemius muscle in response to short‐term resistance and endurance training were maintained despite the presence of a substantial tumour burden and other cancer‐associated physiological impairments. These findings suggest that the capacity for skeletal muscle adaptation to exercise may remain preserved even in the presence of cancer.

Taken together, the contributions in this Special Issue illustrate the translational character of *Exercise as Medicine*, spanning work from mechanistic studies in experimental models to clinical investigations and systematic reviews. This reflects a long‐standing aspiration within physiology to bridge laboratory discovery and clinical practice. In this sense, *Exercise as Medicine* can be viewed not as a new idea but rather as the scientific realisation of an old medical intuition. Already in the sixteenth century, the Spanish physician Cristóbal Méndez wrote ‘if we use exercise under the conditions which we will describe, it deserves lofty praise as a blessed medicine that must be kept in high esteem’ (Berryman, 2010). Today, more than four centuries later, the task of physiology and clinical science is precisely to define those conditions: to determine for whom exercise is indicated, by which mechanisms it exerts its effects, and how it can be applied safely and effectively as a therapeutic intervention.

## AUTHOR CONTRIBUTIONS

Ronan M. G. Berg: Conception, data interpretation, first draft. All authors: revisions and approval of the final version of the manuscript. All authors agree to be accountable for all aspects of the work in ensuring that questions related to the accuracy or integrity of any part of the work are appropriately investigated and resolved. All persons designated as authors qualify for authorship, and all those who qualify for authorship are listed.

## CONFLICT OF INTEREST

Harry Rossiter reports consulting fees from the NIH RECOVER‐ENERGIZE working group (1OT2HL156812) and is involved in contracted clinical research with Biocient, Intervene Immune, Mezzion, Regeneron, Respira and Roche. He is a visiting Professor at the University of Leeds, UK and the University of Pavia, Italy. He reports a patent application filed by The Lundquist Institute, titled ‘Testing System to Diagnose Neuromuscular Deconditioning and Pathologic Conditions’. He receives royalties as a contributor to the textbook *Wasserman and Whipp's Principles of Exercise Testing and Interpretation*.
